# Are There Multiple Motivators for Helping Behavior in Rats?

**DOI:** 10.3389/fpsyg.2020.01795

**Published:** 2020-07-29

**Authors:** Phietica R. R. Silva, Regina H. Silva, Ramón Hypolito Lima, Ywlliane S. Meurer, Bruno Ceppi, Maria Emilia Yamamoto

**Affiliations:** ^1^Laboratory of Evolution of Human Behavior, Federal University of Rio Grande do Norte, Natal, Brazil; ^2^Postgraduate Program in Psychobiology, Department of Physiology and Behavior, Federal University of Rio Grande do Norte, Natal, Brazil; ^3^Behavioral Neuroscience Laboratory, Department of Pharmacology, Federal University of São Paulo, São Paulo, Brazil; ^4^Postgraduate Program in Neuroengineering, Edmond and Lily Safra International Institute of Neuroscience, Santos Dumont Institute, Macaiba, Brazil; ^5^Department of Psychology, Federal University of Paraíba, João Pessoa, Brazil; ^6^Neuroscience and Behavior Laboratory, Department of Physiology and Pharmacology, Federal University of Ceará, Fortaleza, Brazil

**Keywords:** empathy, prosociality, rat, learning, social behavior

## Abstract

Empathy is the ability to (a) be affected by and share the emotional state of another; (b) assess the reasons for the other’s state; and (c) identify with the other, adopting their perspective. This phenomenon has been shown to exist in several species and is proposed as a motivator for prosocial behavior. The experimental study of this feature in laboratory rodents is a more viable alternative in comparison to wild animals. A recent report showed that rats opened a door to free their cage mate from a restraint box. Although this behavior has been suggested to be motivated by empathy, this fact has been questioned by several studies that proposed other motivators for the releasing behavior. In the present study, we use an adaptation of the protocol of releasing behavior to investigate aspects of empathy and pro-sociality such as familiarity and reciprocity. In addition, we addressed some potential motivational factors that could influence this behavior. The main results showed that (1) rats opened the restraint box to free conspecifics most of the time; (2) direct reciprocity or past restriction experience did not improve releasing performance, probably due to a ceiling effect; (3) after a series of trials in the presence of a restricted conspecific, the free rat continues to open the restraint box even if it is empty; (4) in general, the opening performance improves across trials and phases, resembling learning curves; (5) if the first series of trials occurs with the empty box, the opening behavior does not occur and is modest in subsequent trials with a trapped animal; (6) the exploratory drive toward the restraint box and desire for social contact do not seem to function as key motivators for releasing behavior. In conclusion, our findings do not support that the opening behavior is exclusively related to empathic motivation. While multiple factors might be involved, our study suggests that task learning triggered (and possibly reinforced) by the presence of the restricted rat can function as a motivator. Further investigations are required to fully understand the mechanisms and motivation factors guiding the releasing behavior.

## Introduction

Prosocial behavior refers to any action that benefits others, regardless of whether the actor benefits or not from the process (Schroeder et al., 2014). In a wider sense, the term prosocial behavior may include many subcomponents, such as mutualism, altruism, helping, and cooperation (Schroeder et al., 2014). Several factors give support to the development of prosocial strategies. The role of empathy as a motivator for prosocial behavior has been a focus of studies over the last decade. Empathy is the ability to (a) be affected by and share the emotional state of another; (b) assess the reasons for the other’s state; and (c) identify with the other, adopting their perspective (de Waal, 2008). In other words, the term entails emotional as well as cognitive reactions of an individual when observing the experience of another individual (Preston and de Waal, 2002; Panksepp and Lahvis, 2011; Shamay-Tsoory, 2011). From the evolutionary point of view, empathy may have favored prosocial behavior in social life, inhibiting aggression and facilitating cooperation among members of social groups. It is a skill common to humans and other animals, which evolved primarily to support a wide range of prosocial behaviors, ranging from parental care to helping behavior (Decety et al., 2016).

A cross-species review pointed out five major attributes of empathy: familiarity, similarity, learning, past experience (of the situation of distress, for example), and salience (Preston and de Waal, 2002). Given the greater probability of repeated interactions in a particular group, the effect of familiarity may favor direct helping to members of the group itself. Accordingly, when laboratory rats are housed in the same cage, they increase the chances of helping each other, even when they belong to different strains (Ben-Ami Bartal et al., 2014).

The neurobiology involved in the phenomenon of empathy is not completely understood. However, it is known that corresponding mirror neurons fire when people see others performing a certain action (Decety and Ickes, 2009). Neuroimaging studies in humans show that the neural network involved in the first-hand experience of pain also responds to the suffering of another individual (Jackson et al., 2005, 2006; Lamm et al., 2007; Decety et al., 2010, 2012). In addition, neuroendocrine mechanisms are involved in social behavior. The hormone oxytocin, for example, increases the accuracy of emotion recognition (Domes et al., 2007), trust (Kosfeld et al., 2005), generosity (Zak et al., 2007), and cooperation (De Dreu et al., 2010) in humans.

In order to understand neurobiological mechanisms related to empathy, we can study basic forms of empathy in non-human animals (Panksepp and Panksepp, 2013; Decety et al., 2016). To achieve this purpose, the experimental study of behavior in laboratory rodents is a more viable alternative in comparison to the study of wild animals. Laboratory strains of rats and mice have an extensive documented experimental history (Panksepp and Lahvis, 2011), low-cost breeding, and relatively easy maintenance. However, the assessment of prosocial behavior in these animals is not usual in the literature, and there is still an ongoing debate as to whether they are capable of any form of empathy (Vasconcelos et al., 2012; Silberberg et al., 2014).

On the other hand, there are studies that support the investigation of physiological and pathological aspects of empathy in laboratory rodents (for review see Wrighten and Hall, 2016). For example, in mice, there are clear signs of emotional contagion (Langford et al., 2006; Guzmán et al., 2009; Bruchey et al., 2010; Jeon et al., 2010; Kim et al., 2012; Nowak et al., 2013). Various strains of rats are able to express helping behavior (Church, 1959; Rice and Gainer, 1962; Sato et al., 2015). One of the earliest studies on empathy-motivated prosocial behavior in rodents has shown that rats refrain from pressing a bar to receive food in order to avoid electrical shocks to a conspecific (Church, 1959). Moreover, rats exposed to a painful stimulus showed increased fear to the pain of others (Church, 1959). Rats also press a bar to lower a conspecific suspended in air by a harness (Rice and Gainer, 1962).

A more recent report showed that rats opened a door to free their cage mate from an acrylic restraint box. This behavior has been suggested to be motivated by empathy. Rats opened the restraint box less often when the box was empty, or when the conspecific was replaced by an object (Ben-Ami Bartal et al., 2011). Using the same protocol, researchers found that rats opened the door for both a cage mate and a strange conspecific, but they do not help rats from another strain unless they have lived in the same cage (Ben-Ami Bartal et al., 2014). In a similar experiment, rats released a soaked cage mate from a water area by opening the door to allow the trapped rat into a safe area. When forced to choose between a cage mate and a box with food reward, rats chose to help the cage mate before obtaining food (Sato et al., 2015).

There is evidence that reciprocity (Rutte and Taborsky, 2008) and quality of help received (Dolivo and Taborsky, 2015) can influence prosocial behavior in rats. Rutte and Taborsky (2008) showed that rats are more helpful toward a partner from which they had received help before than a partner that had not helped or a newer partner. These results suggest that direct reciprocity produces higher cooperation predisposition than generalized reciprocity.

Taken together, these studies showed evidence of empathy-motivated prosocial behavior in rats. On the other hand, the demonstration of emphatically motivated behavior in non-human animals has been an issue of controversy, at least in the absence of painful or fear-inducing situations (Silberberg et al., 2014). Furthermore, some authors claimed that other motivational factors could be involved in the releasing behavior reported by Ben-Ami Bartal et al. (2011) (Vasconcelos et al., 2012; Silberberg et al., 2014), while others have interpreted the given behavior as empathy-elicited prosociality (De Waal, 2012; Decety et al., 2012).

Nevertheless, it is clear that there is a need for more elements that relate releasing behavior to empathy and prosociality in rats if one intends to establish this behavior as a protocol suited for the study of these phenomena. In the present study, we use an adaptation of the protocol of releasing behavior proposed by Ben-Ami Bartal et al. (2011) to investigate aspects of empathy and prosociality such as familiarity and reciprocity. In addition, we addressed some potential motivational factors that could influence this behavior. Specifically, we investigated (1) whether the presence of a trapped rat motivates a free one to act prosocially, (2) whether the previous trapped rat becomes a liberator when the roles in the test are changed (to test the influence of past experience), (3) whether rats that had been helped before would liberate an unfamiliar rat, (4) whether the rats express the same releasing behavior in absence of the conspecific, and (5) a possible role of other motivators (non-empathic) for opening behavior.

## Materials and Methods

### Subjects

Fifty-two male Wistar rats aged 3–4 months were housed under a 12/12-h light-dark cycle, with unrestricted access to food and water. Rats were allocated in pairs 2 weeks prior to the experiments to familiarize themselves with their partner and the new cage environment. During these 2 weeks, all animals were handled daily for 1 min each. All procedures were carried out according to the Brazilian rules for the use of animals in scientific research (Law No. 11.794) and approved by the local ethics committee (CEUA/UFRN No. 021/2015).

### Apparatus and General Procedures

**FIGURE 1:**
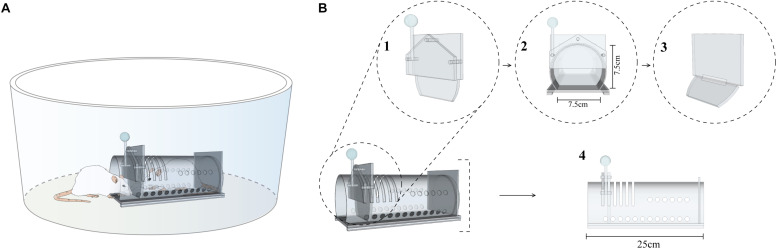
Schematic representation of the apparatus. **(A)** Arena with restrain box and subjects. **(B)** First door (B1); frontal view of the first door (B2); second door (B3); and restrain box (B4).

The tests were performed in a circular open field made of wood and covered with white formica (Figure 1; 62.5 cm in diameter and the surrounding wall is 31.5 cm high). In the center of the arena we placed an acrylic restraint box (25 × 7.5 × 7.5 cm). The box had small ventilation holes and a door that could only be opened from the outside by applying enough force downward on an attached lever (Figure 1). A slide door was added to the set in Experiments 2 and 3 (see below). This was a slide door that only swayed when pushed from the inside, precluding the reentrance of the subjects after they exit the restraint box. The behavior of the animals was recorded by a webcam placed over the apparatus and analyzed by a video-tracking software (any-maze, Stoelting Co., United States). Only the free rat was tracked. In order to generate contrast with the bottom of the arena, we placed a black marking on the back of the free rats. The apparatus environment was surrounded by a black curtain with four focal soft lights at the top of each corner, corresponding to 150 lux at the arena floor level.

The behavioral parameters that were registered were latency (in seconds) to open the restraint door, opening rate [percentage of animals that opened the restraint box in a trial), and percentage of time in social interaction after release (time spent in social interaction (s)/remaining time of session after release (s) × 100]. Social interaction was registered (duration in seconds) when the animals approached each other and at least one of them had the vibrissae in physical contact with the other rat. Time spent in active (sniffing, following, walking over, or under the partner) or passive (when they lay next to each other) interactions were counted together. The social interaction evaluation was conducted as proposed by previous studies (File and Hyde, 1978; Calzavara et al., 2011).

Before the beginning of the experiments, rats underwent daily habituation sessions, where they were free to explore the arena and restraint box without the door. At the beginning of each habituation day, rats were brought to the test room where they remained in their cages at least 15 min before the session. Rats were habituated in pairs for 30 min each day. Careful cleaning of the apparatus was performed between sessions with 5% (v/v) ethanol solution.

### Experimental Design

Experiments and phases described below are summarized on Table 1.

**TABLE 1:** Summary of the phases in each of the three experiments.

Experiment 1 (*n* = 20/10 pairs)	Experiment 2 (*n* = 16) (addition of a second door) (*n* = 16/8 pairs)	Experiment 3 (control) (*n* = 8 per group)
1. Releasing task	1. Releasing task	Control – empty box (sample 1)
2. Direct reciprocity	2. Direct reciprocity	Control – toy rat (sample 2)
3. Generalized reciprocity	3. Generalized reciprocity	1 – Releasing task (half sample 1 and half sample 2)
4. Empty restriction box	4. Empty restriction box	

#### Experiment 1

This experiment was conducted in four phases. Each phase comprised of 12 daily sessions that lasted 30 min each. The animals selected for trapping were kept in the restriction box for 2 min prior to the introduction of the free rat. Ten pairs of naïve rats participated in this experiment.

##### Phase 1: Releasing task

The test started when the free rat was placed in the experimental arena. After the release of the trapped rat, the animals remained in the arena until the end of the session. If by the end of the 30-min session the rat had not opened the door of the restriction box, the experimenter would open the door, allowing the rats to interact for 10 min.

##### Phase 2: Direct reciprocity

To evaluate direct reciprocity, the rats that participated in Phase 1 were submitted to the same procedure, but in reversed roles. Again, the tests were repeated for 12 days, following the same procedures of Phase 1.

##### Phase 3: Generalized reciprocity

The same rats participated in Phase 3, in order to investigate helping between unfamiliar subjects (generalized reciprocity). At this stage, a rat from a cage was paired with a rat from another cage. The tests were repeated for 12 days, following the same procedures of the previous phases.

##### Phase 4: Empty restriction box

Eight rats with the best opening scores in the previous phases were selected to undergo the test without the presence of a trapped conspecific. The selection was applied to avoid facilitation of behavioral extinction. The rats were exposed to the arena, with an empty restriction box, in 12 daily sessions of 30 min each.

#### Experiment 2

In this experiment, a second door was added to prevent rats from entering the restraint box after the release (Figure 1). The purpose of this experiment was to investigate if entering the box could be a motivational factor for releasing behavior. The top-hung second door allowed the passage only from the inside. In this experiment, eight pairs of naïve rats were used, and all procedures were held identically to Experiment 1.

#### Experiment 3

Tests were performed with the same apparatus, with the two doors in the restraining box, and animals went through the same handling, habituation, and testing conditions, except that there was no trapped animal. In the Empty Box Control, rats were tested individually with an empty restraint box. In the Toy Control, a toy rat made from biscuit was used as object condition control. The rationale for this condition is to control the experiment for novelty within the restraint box.

In both the Empty Box and Toy Controls, the animals (*n* = 8 naïve rats in each control) had no previous experience with the task. After the usual 12 daily 30-min sessions, we used the same animals to perform a releasing task phase, similar to Phase 1 of the first experiment, in which half of the rats from the Empty Box and Toy Controls became the free rat and the other half became the trapped rat. For the purpose of comparison with the other experiments, we will call the phase with the releasing task in Experiment 3 “Phase 1.”

### Statistical Analysis

All data were checked for normality using the Kolmogorov–Smirnov test. We had some technical problems in the recording of Phase 2 of Experiment 1, and for this reason only part of the results of that phase were included in the analysis. Opening rates and percentage of social interaction were analyzed by one-way ANOVA followed by Tukey’s test. Mean opening latency (for each animal in the trials of a phase) was analyzed by Kruskal–Wallis analysis of variance and Mann–Whitney *U* test when necessary. In Experiments 1 and 2, the described analyses were initially conducted considering Phases 1–3. Afterward, another set of analyses was conducted including Phase 4, in which subjects were selected based on the performance on previous phases. In this set of analyses, only animals that went through Phase 4 were included, and their performances on Phase 4 were compared to the phase of their first experience as a liberator (Phase 1 or 2). Latency to open across trials within a phase was analyzed by Friedman’s test for related samples. Pearson’s test was applied to check a possible correlation between social interaction and opening performance. All results were displayed as mean ± standard error (SE) and *p* = or < 0.05 was adopted as the level of statistical significance.

## Results

### Opening Rates

**FIGURE 2:**
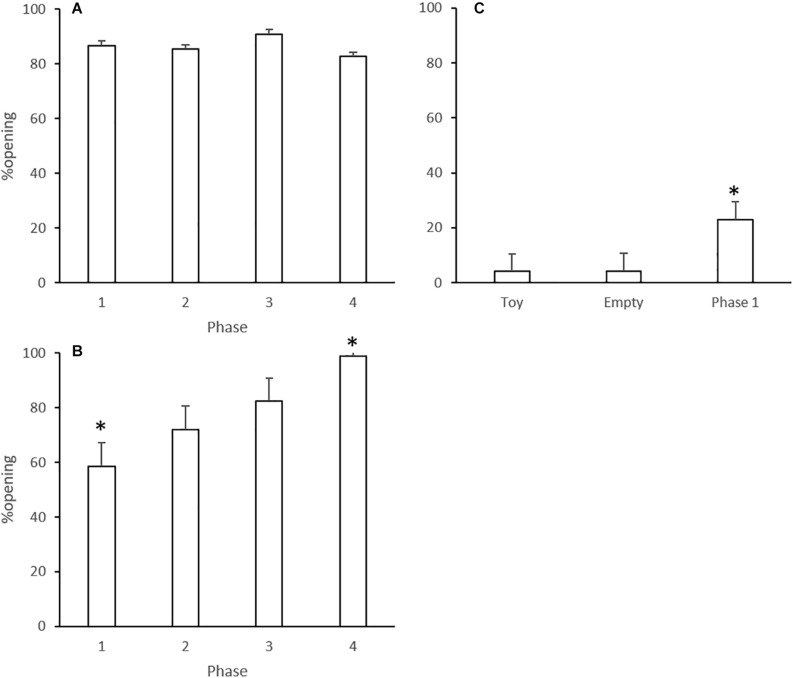
Opening rates (percent of animals that opened the restraint box in each experimental phase) in Experiments 1 **(A)**, 2 **(B),** and 3 **(C)**. Data are mean + SE. ^∗^*p* < 0.05 compared to the other phases of the same experiment. One-way ANOVA followed by Tukey’s test.

The percentage of animals that showed an opening behavior is displayed in Figure 2. In Experiment 1, most of the rats opened the restraint box in all phases, and one-way ANOVA did not show significant differences among Phases 1–3 [*F*(2,27) = 1.13; *p* = 0.339] (Figure 2A). Opening rates by rats that underwent Phase 4 did not differ from previous sessions (*p* = 0.364).

In Experiment 2, one-way ANOVA considering Phases 1–3 revealed an effect of phases [*F*(2,33) = 14.04; *p* < 0.001]. Tukey’s post-test showed that the animals presented the lowest opening rate in Phase 1 (*p* < 0.01, Figure 2B). Opening rates by rats that underwent Phase 4 were increased compared to previous sessions (*p* = 0.364).

One-way ANOVA also showed an effect of phases for Experiment 3 [*F*(2,33) = 19.391; *p* < 0.001]. Tukey’s post-test revealed that the opening rate in Phase 1 was significantly increased compared to toy and empty box phases (*p* < 0.001 for both comparisons, Figure 2C).

### Latency for Opening

Latency curves with mean values in each trial in all the phases from Experiments 1, 2, and 3 are shown in Supplementary Figures S1–S3. In summary, latencies for opening the restraint door were low across trials in Experiments 1 and 2, even in the trials with the empty box (Phase 4 of these experiments). In Experiment 3, as the opening rate was lower compared to the other experiments (see above), the latencies were high across trials with the toy or empty box and were slightly decreased in Phase 1 (with the trapped conspecific). These data are in accordance with the analysis for the mean phase latency for each animal, which is described below.

**FIGURE 3:**
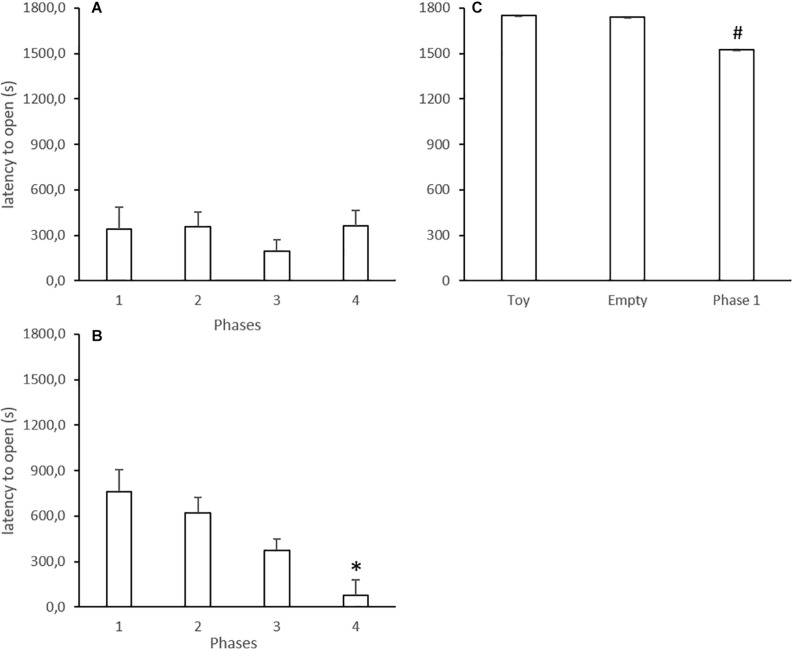
Mean latencies (in seconds) to open the restrain box in experimental phases of Experiments 1 **(A)**, 2 **(B),** and 3 **(C)**. Data are mean + SE. ^∗^*p* < 0.05 compared to Phases 1 and 2 within the experiment. #*p* < 0.05 compared to Phases 1 from Experiments 1 and 2. Kruskal–Wallis analysis of variance followed by Mann–Whitney *U* test.

The mean latencies in each phase are displayed in Figure 3. In Experiment 1, the Kruskal–Wallis test did not reveal differences when Phases 1–3 were compared within the experiments (*H* = 2.00, *p* = 0.368; Figure 3A). Latency for opening by rats that underwent Phase 4 did not differ from previous sessions (*p* = 0.600).

In Experiment 2, the Kruskal–Wallis test did not reveal differences when Phases 1–3 were compared within the experiments (*H* = 4.94, *p* = 0.085; Figure 3B). However, opening latency by subjects that underwent Phase 4 was lower compared to their performance in the previous phase as a liberator (*p* = 0.001).

In Experiment 3, the Kruskal–Wallis test did not reveal differences between phases within the experiment (*H* = 4.51, *p* = 0.105; Figure 3C).

Additionally, we compared similar phases between experiments, and the analysis revealed that latency values in Phase 1 in Experiment 3 were significantly higher compared to Phase 1 in Experiments 1 (*U* = 4.00; *p* = 0.001) and 2 (*U* = 8.00; *p* = 0.010).

### Social Interaction

**FIGURE 4:**
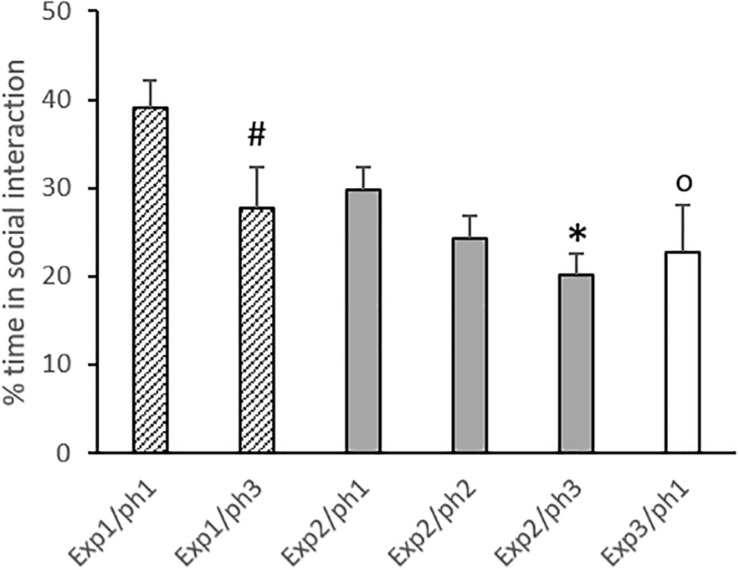
Percent time spent in social interaction (considering the remaining time of the session after releasing) in phases (ph) of experiments (exp). Data are mean + SE. #*p* = 0.05 compared to Phase 1 in the same experiment. ^∗^*p* < 0.05 compared to Phase 1 in the same experiment. ^O^*p* < 0.05 compared to Phase 1 in Experiment 1. One-way ANOVA followed by Tukey’s test.

Only trials in which the trapped animal was released were considered for this analysis. Phase 2 from Experiment 2 was excluded from the analysis due to the previously mentioned technical problems. Results are displayed in Figure 4.

One-way ANOVA revealed that the percentage of time spent in social interaction was different between phases and experiments [*F*(5,43) = 4.20; *p* = 0.003]. For Experiments 1 and 2, the Tukey *post hoc* test showed that animals in Phase 3 presented reduced social interaction when compared to Phase 1 (*p* = 0.05 and 0.03, respectively). Phase 1 from Experiment 3 was compared to Phase 1 of the other experiments, and a significant difference was detected in relation to Experiment 1 (*p* = 0.03).

### Correlation Test

Pearson’s test applied to mean opening latency vs. percentage of time in social interaction did not reveal a significant correlation (*R* = −0.101; *p* = 0.495).

## Discussion

The design of Phase 1 of the first experiment was similar to the protocol established by Ben-Ami Bartal et al. (2011), in which rats opened a door to release a trapped cage mate without receiving previous training or any direct reward. According to that study, the opening behavior was motivated by empathy. In other words, the free animal identified the uncomfortable condition of the cage mate and proceeded to release the conspecific. In the original study, most rats learned to open the door of the restraint box and free the conspecific around the sixth day of testing. In the present study, the rats began to open the door of the restraint box on the first day of testing, and kept opening it across all sessions, resulting in a high opening rate and low mean latency (Figures 2A, 3A). Despite the different pattern of the opening behavior learning curve, these results corroborate the findings of Ben-Ami Bartal et al. (2011).

As previously mentioned, the rats in our study were faster when performing the opening task than those from the Ben-Ami Bartal et al. (2011). This result may be due to the use of different rat strains. Wistar rats (used in the present study) are more active than the Sprague–Dawley rats that were used in the previous study (Schmitt and Hiemke, 1998; López-Rubalcava and Lucki, 2000; Clemens et al., 2014). In this respect, the higher exploration of the environment in a shorter space of time may lead to a faster resolution of an exploratory task (Hughes, 1997). Another possible explanation could be the shape of the arena. While the arena used herein is circular, Ben-Ami Bartal and colleagues used a square arena, which might have induced the animals to stay longer in the corners due to defensive behavior (Gould et al., 2009).

In Phase 2, we tested the influence of experience concerning the releasing behavior. The rats that were restricted in Phase 1 became the free ones in Phase 2. We hypothesized that the unpleasant experience of imprisonment in the previous phase would favor the releasing behavior in Phase 2, because empathy can be reinforced by experience in a situation of distress (Preston and de Waal, 2002). Our results showed that the opening rate and mean latency in the initial 6 days of Phase 2 were similar to Phase 1 (Figures 2A, 3A). In other words, we did not see the expected incremental effect of experience. This was probably due to a ceiling effect, i.e., the high performance of the rats in Phase 1 made observing any behavioral improvement difficult.

In Phase 3, we paired rats that had no previous contact with each other, but that had already experienced both free and restricted situations. The results showed that regardless of the unfamiliarity, the animals sustained the opening behavior and had a low mean opening latency (Figures 2A, 3A). This result also corroborates the previous work showing that unfamiliar rats of the same strain sustain the prosocial behavior, while rats from different strains are more resistant to behave likewise (Ben-Ami Bartal et al., 2014).

In summary, Phases 1–3 demonstrated that rats do release the conspecific that is trapped in the restriction box, corroborating previous studies (Ben-Ami Bartal et al., 2011, 2014). In addition, we extend those results in the way that reciprocity and familiarity do not seem to favor the prosocial behavior, although a ceiling effect might take place regarding the opening performance.

In Phase 4, experienced free rats were exposed to the empty restraint box inside the arena. If the motivation to open the restriction box is based on empathic features, we would expect that the animals would not open the box, or at least would show decreased opening rates and/or increased latencies to open it. Surprisingly, however, rats sustained the opening behavior even in the absence of a conspecific, and with similar opening rates and latencies as in the other phases (Figures 2A, 3A). In this way, we could not confirm that the prosocial behavior was driven by empathic motivation.

Beyond the persistence in opening the door even in the absence of a conspecific, there are other factors that could be associated with the motivation to do so. For example, during Experiment 1, we observed that the free rats, after releasing the trapped animal, entered the restraint box. We hypothesized that exploring the restraint box would motivate the animals to open the box because of novelty or the possibility of a “safe place” within the open arena.

To confirm this outcome, we reanalyzed all videos to register how many rats entered the restraint box after opening. In fact, there was a high entry rate after opening at all stages (around 80% of the animals in each phase of Experiment 1, Supplementary Figure S4). The entry was counted when animals entered the restraint box within 10 s after the exit of the trapped rat.

The restraint box should be an aversive environment, as animals inside it are considered confined and require help in order to be released (Ben-Ami Bartal et al., 2011). However, it is well known that rodents prefer closed environments, dens, and corners, where they can touch the surrounding with their vibrissae (Cardenas et al., 2001). Open environments represent greater predator exposure and cause anxiety (Treit et al., 1993). Thus, we suggest that the box could be working as a shelter inside the arena, leading to a preference for the restraint box, which would motivate the opening behavior. This suggestion is corroborated by a study that uses a similar protocol with mice and found that the animals performed the releasing behavior because they were interested in the restraining tool (Ueno et al., 2019).

Therefore, we did not find clear evidence of empathy-motivated prosocial behavior in Experiment 1, mainly because we observed a high opening rate throughout all phases (Figure 3A), including the one without a trapped conspecific. As previously mentioned, this outcome suggests that opening the restraint box is a defensive or escape behavior, motivated by fear/anxiety, and directed to a possible safe area inside the arena. To test this hypothesis, we designed a second experiment with an adaptation in the restraint box.

In Experiment 2, the entrance of the free rat into the restraint box was precluded by the presence of a second door. In Phase 1, the opening rate was lower than that observed in the same phase of Experiment 1. Nevertheless, the opening rate increased from 60% (Phase 1) to almost 100% by Phase 4 (Figure 2B). Accordingly, the mean latency to open decreased progressively, and the lowest values were found in Phase 4 (Figure 3B). These data have two relevant outcomes. First, the blockage of the restraint box did not interfere with the motivation to open it, i.e., the free animal still performed the releasing behavior despite that there was no possibility of entering the restraint box. Second, similar to that which happened in Experiment 1, the motivation to open the restraint box was not diminished in the absence of the trapped conspecific. In fact, the contrary was found; Phase 4 displayed the highest opening rate and the lowest opening latency.

Thus, although prosociality cannot be ruled out as a motivator to the opening behavior showed in the present study, we suggest that there is at least one other factor implicated in the drive to open the restraint box. In this way, there seemed to be a learning process throughout the behavioral sessions in the experiments. Indeed, the analysis of the opening rate and mean opening latency across phases of Experiment 2 showed a clear learning curve (Figures 2B, 3B). This profile was not clear in Experiment 1 when the mean value of the phases was analyzed, perhaps because the opening rate of Phase 1 was already high. Nevertheless, the decrement in latency to open across trials in most phases of the study (Supplementary Figures S1, S2) suggests that there was a learning-related component, i.e., there was an improvement in the performance of the animal across repeated trials.

Learning the task might have worked as a motivator. In other words, due to the multiple exposures to the environment, animals seemed to improve their capacity to discriminate objects and tasks within the arena. This behavioral change could be related to “perceptual learning” or priming (Squire and Dede, 2015). Even in the presence of the second door (Experiment 2), rats had increasing opening rates and decreasing latency for opening across the experiment. We suggest that there is a role of experience in the execution of the releasing behavior, irrespective of an empathic/prosocial motivation. To this end, we performed a third experiment to investigate behavioral persistence as an effect of a high opening rate history.

In Experiment 3, animals from the Empty Box Control presented a low opening rate, pointing out the need of a trapped conspecific to motivate the opening behavior (Figure 2C). Animals from the Toy Control also displayed a low opening rate, showing that the presence of an object simulating a rat was not enough to trigger the opening of the restraint box (Figure 2C). Accordingly, the mean latencies of these two groups were very high, reaching the maximum value of session duration (Figure 3C). Interestingly, when we used the same rats for further 12 days of testing under the same conditions of Phase 1 of the other experiments (with the presence of a trapped cage mate), there was still a low opening rate and high mean latency, although the opening performance was improved compared to the controls (Figures 2C, 3C).

Comparing the results of the three experiments, we noted that the order of stimulus presentation is relevant to the occurrence of releasing behavior. When the first series of contact with the task environment was with a trapped conspecific, the rats demonstrated high opening rates and low opening latencies, even when the restrained rat was no longer present (Experiments 1 and 2). Conversely, the presence of the trapped conspecific after several expositions to an empty box or to a toy rat hindered the expected increase in the opening rate (Experiment 3). In other words, the presence of the trapped animal was relevant to engage the rat in opening behavior. It remains unknown if this fact represents an empathic motivation. Nevertheless, we might hypothesize that changes in the emotional state of animals (elicited by the presence of a trapped conspecific) may be a modulatory factor triggering helping behavior. This view implicates the role of a learning process in the performance of releasing behavior, in the sense that emotional stimuli (presence of the trapped rat) facilitate or even promote the learning of tasks (Squire and Dede, 2015). In this context, the opening performance would be better with the repeated exposition in the presence of the conspecific, regardless of motivation.

In summary, the results from the three experiments suggest that there is a learning-associated opening behavior, but it needs to be triggered by an initial stimulus (presence of the conspecific) and possibly reinforced by the release of the rat in each session. At the same time, the occurrence of an empathically motivated prosocial behavior cannot be ruled out, as the presence of the trapped rat during the first expositions to the environment is determinant toward performing the task (opening of the restraint box). Additionally, with multiple expositions to a certain environment, novelty-stimulated exploratory activity diminishes, but the opening behavior remains.

In light of these observations, there is the possibility that our results are explained by social facilitation of learning, i.e., the beneficial effect of the presence of a conspecific on task performance (Zajonc, 1965). Types of social learning are well recognized as basic aspects of behavior relevant for animal cooperation and community life (for review see: Geen and Gange, 1977; Reznikova and Panteleeva, 2008; Galef, 2013). Social facilitation of learning has been described for several species (Bond and Titus, 1983; Zion et al., 2007; Lipina and Roder, 2013; Demolliens et al., 2017; Lau et al., 2019), including laboratory rats (Gardner and Engel, 1971; Becker and Franks, 1975; Varlinskaya and Spear, 2009; Gipson et al., 2011; Dorfman et al., 2016). Hollis and Nowbahari (2013), Hollis et al. (2015), and Turza et al. (2020) investigated conspecific rescue behavior in ants, suggesting that sharing the environment with predators facilitates the expression of these behaviors, although non-rescuer species also seem able to display these. In this study, the presence of a trapped conspecific probably played the role of facilitation.

Neurobiological correlates of this phenomenon are rare, but there is evidence of neural networks specific to learning processes under social contexts (Demolliens et al., 2017). Further, oxytocin, a hormone linked to empathy and prosocial behavior (Stetzik et al., 2018), has been shown to facilitate learning with social feedback (Hu et al., 2015). Interestingly, the administration of oxytocin improves helping behavior in rats, and this effect is dependent on social context (Yamagishi et al., 2019). Thus, it is possible that the protocol of releasing behavior, projected to model empathy in rodents, involves a complex relationship between empathy and social facilitation of learning the task of opening the restraining box.

Finally, as mentioned previously, the empathic motivation to perform this behavior is still widely debated. Literature points out the seeking of social contact as a possible motivation to the releasing behavior, although not a determining factor for opening (Silberberg et al., 2014). If this were true, we would expect a correlation between the performance of releasing behavior and the social interaction that followed the release. In the present study, we evaluate the amount of social interaction that occurred after the release, and the data showed no correlation with the opening performance. In addition, in Experiments 1 and 2 the amount of social interaction decreased across phases (Figure 4), while the opening performance usually improved. If the aim of performing the releasing behavior were social contact, we would expect a progressive increase in social interaction across phases. Conversely, it is possible that this decrease in social interaction with repeated exposure is due to habituation, or loss of interest in the conspecific (Panksepp and Beatty, 1980). Once again, if the social contact were the motivator for the releasing behavior, the loss of interest in social interaction would inhibit the opening performance. In addition, in Phase 1 of the third experiment, the animals showed a similar amount of social interaction as in the other phases/experiments (except for Phase 1 of the first experiment), while showing a poor opening performance compared to animals in the other experiments. This evidence suggests that, at least under our experimental conditions, we can rule out the desire for social contact as a determining motivator for the releasing behavior.

Regarding a possible limitation of this study, audible or ultrasonic vocalizations are suggested as possible triggers for helping behavior in rodents, as these evoke emotional contagion (Saito et al., 2016). In the case of the releasing behavior protocols, the free rat could open the box in order to stop the alarm calls of the conspecific (Schwartz et al., 2017). In the present study, there were no audible vocalizations during the periods of restraint, and we did not register possible ultrasonic vocalizations. Although this is a limitation of the study, the literature does not seem to support this hypothesis. In the original study that proposed the task, Ben-Ami Bartal et al. (2011) reported that the vocalizations were not frequent enough to be considered motivators for releasing behavior, and occurred more often in the initial sessions, when the opening rates were low. Furthermore, studies with pain-related emotional contagion have pointed out that, although being a classical response to stressful situations (Sanchez, 2003), ultrasound vocalizations are not the main trigger of vicarious behavior (Atsak et al., 2011).

## Conclusion

In conclusion, there are multiple factors involved in the motivation to the opening behavior in the protocol proposed by Ben-Ami Bartal et al. (2011) and used in the present study. This outcome is relevant as there is an increasing number of studies that used the original or modified versions of the releasing task with the purpose of studying empathy or prosocial behavior in rodents (Ben-Ami Bartal et al., 2014, 2016; Silberberg et al., 2014; Hachiga et al., 2018; Kandis et al., 2018; Karakilic et al., 2018; Fontes-Dutra et al., 2019; Yamagishi et al., 2019). Our study provided data that ruled out the desire for social contact or the exploration of the restraint box as key motivators, at least under our experimental conditions. However, our findings do not support that the opening behavior is exclusively related to empathic motivation, as these suggest a strong role of emotion-related learning in the performance of the task. Further investigations are required to fully understand the mechanisms and motivation factors guiding the releasing behavior.

## Data Availability Statement

The datasets generated for this study are available on request to the corresponding author.

## Ethics Statement

The animal study was reviewed and approved by Comissão de Ética no Uso de Animais – Universidade Federal do Rio Grande do Norte.

## Author Contributions

RS and MY designed and coordinated the study. PS performed the experiments. RS, BC, and YM analyzed the data. PS, RL, and RS wrote the manuscript. All authors contributed in data discussion and in the final manuscript.

## Conflict of Interest

The authors declare that the research was conducted in the absence of any commercial or financial relationships that could be construed as a potential conflict of interest.

## Supplementary Material

FIGURE S1 Latencies to open the restrain box across trials in each phase of Experiment 1. Data are mean + SE. Friedman’s test showed effect of repeated trials for Phases 1 (χ^2^ = 30.93; *p* = 0.001) and 2 (χ^2^ = 11.46; *p* = 0.043).

FIGURE S2 Latencies to open the restrain box across trials in each phase of Experiment 2. Data are mean + SE. Friedman’s test showed effect of repeated trials for Phase 4 (χ^2^ = 21.42; *p* = 0.029).

FIGURE S3 Latencies to open the restrain box across trials in each phase of Experiment 3. Data are mean + SE. Friedman’s test did not reveal effect of repeated trials in any phase.

FIGURE S4 Percentage of entrance in the restrain box after opening in Experiment 1. Data are means + SE.
