# Supplementary figures and images for: Are There Multiple Motivators for Helping Behavior in Rats?
Source: Front Psychol. 2020 Jul 29;11:1795. doi: 10.3389/fpsyg.2020.01795 (PMC7403447; doi:10.3389/fpsyg.2020.01795)

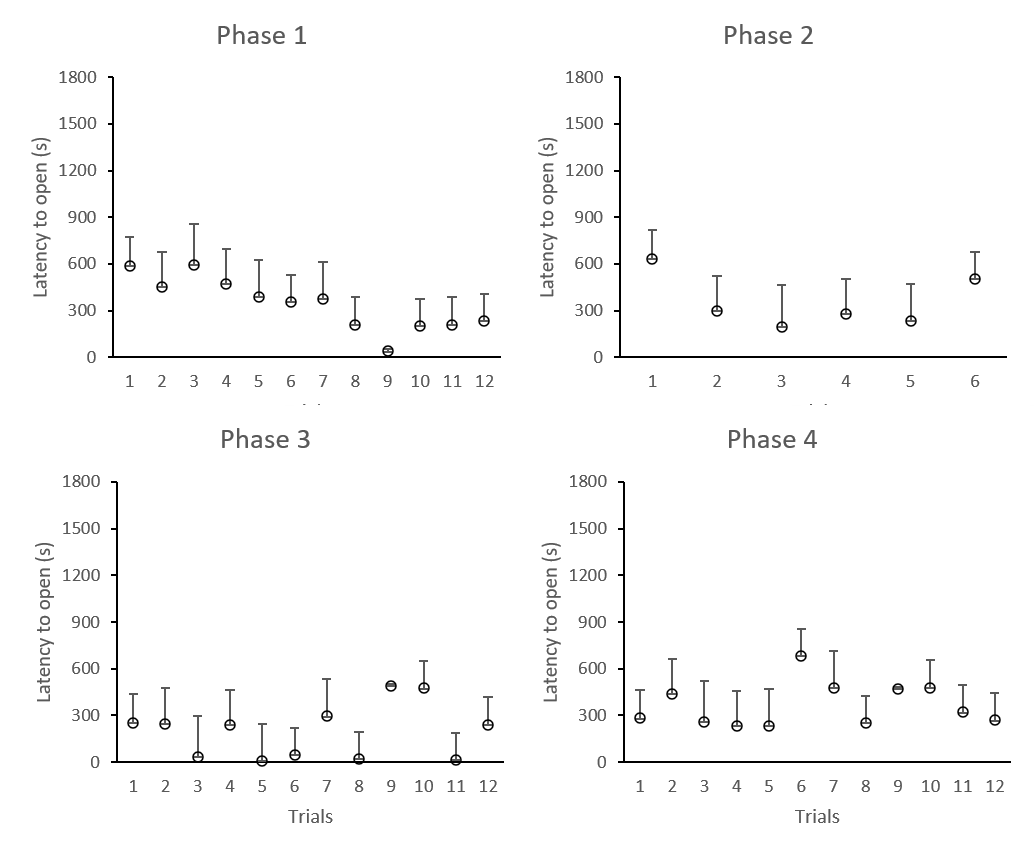

Supplement: FIGURE S1 — Latencies to open the restrain box across trials in each phase of Experiment 1. Data are mean + SE. Friedman’s test showed effect of repeated trials for Phases 1 (χ2 = 30.93; p = 0.001) and 2 (χ2 = 11.46; p = 0.043). [file Image_1.TIF]

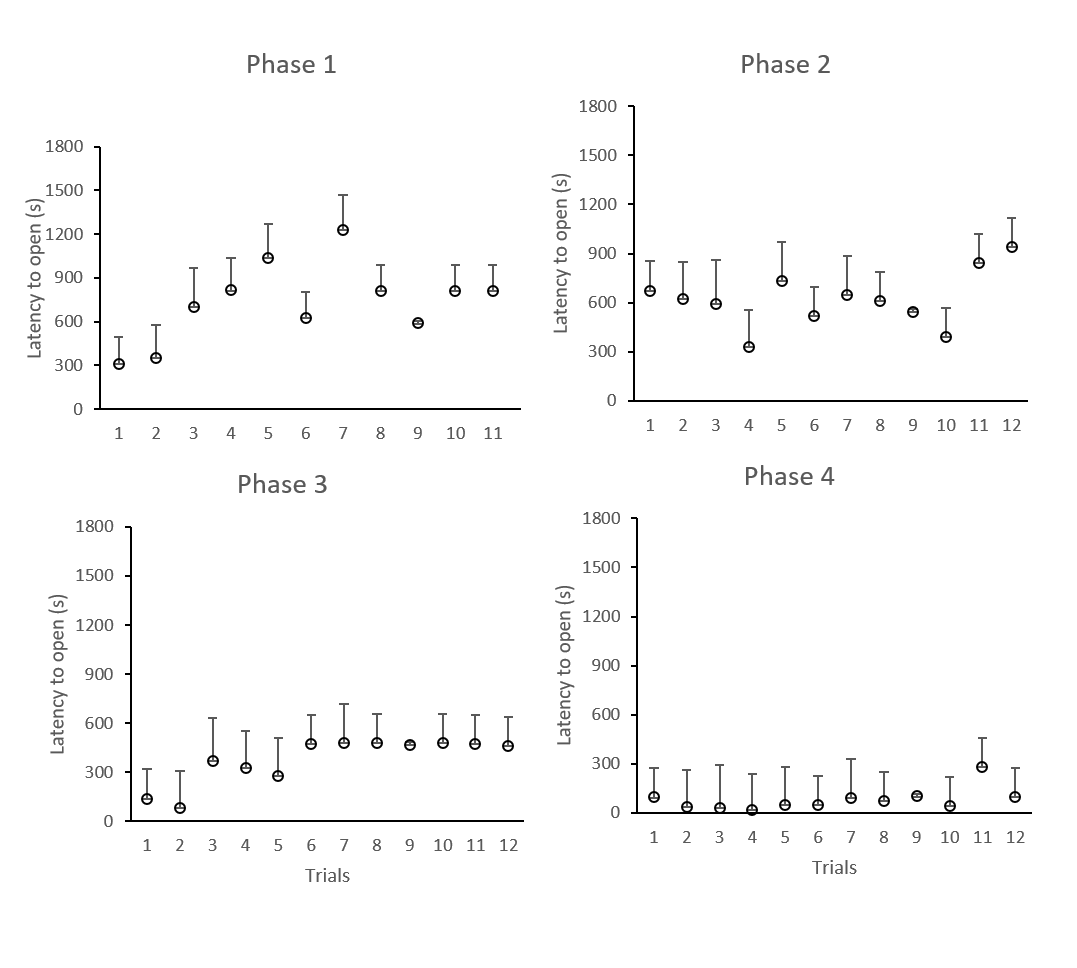

Supplement: FIGURE S2 — Latencies to open the restrain box across trials in each phase of Experiment 2. Data are mean + SE. Friedman’s test showed effect of repeated trials for Phase 4 (χ2 = 21.42; p = 0.029). [file Image_2.tif]

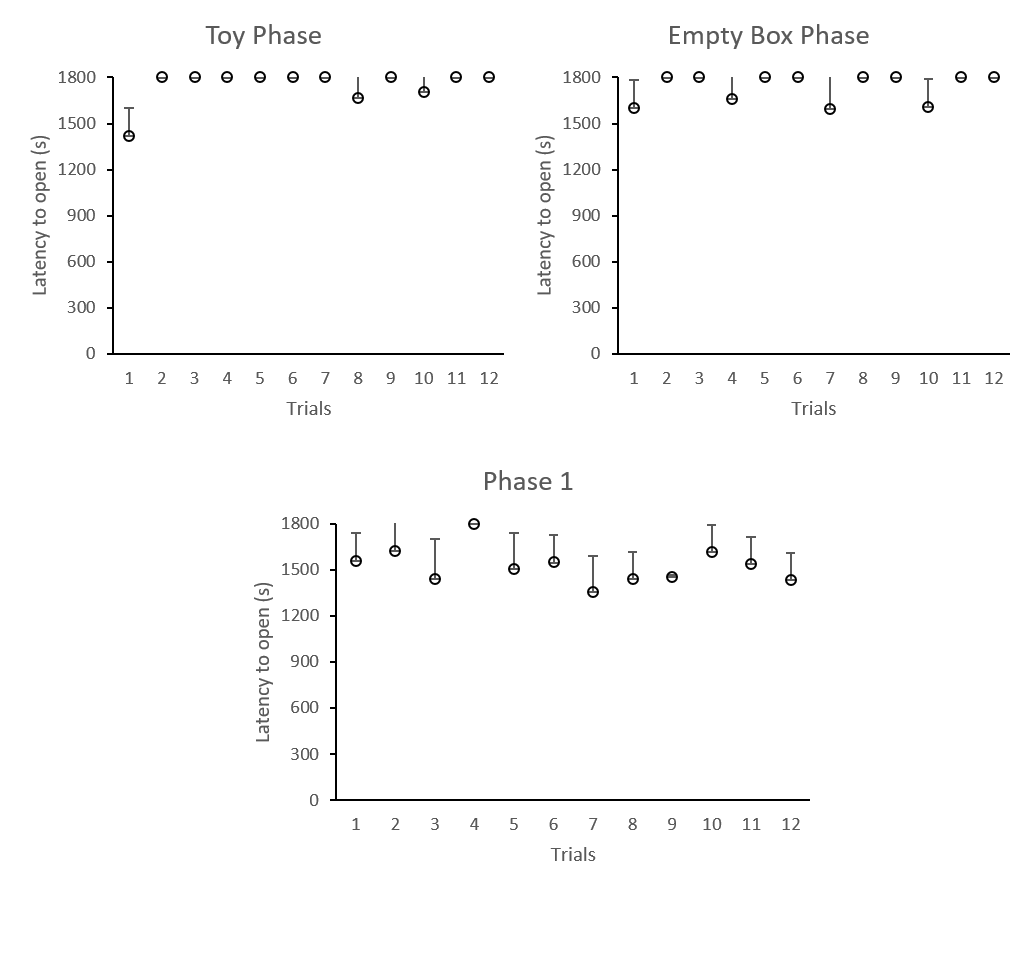

Supplement: FIGURE S3 — Latencies to open the restrain box across trials in each phase of Experiment 3. Data are mean + SE. Friedman’s test did not reveal effect of repeated trials in any phase. [file Image_3.tif]

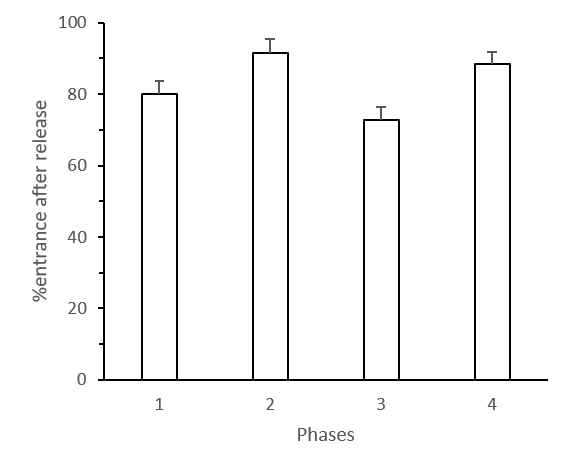

Supplement: FIGURE S4 — Percentage of entrance in the restrain box after opening in Experiment 1. Data are means + SE. [file Image_4.TIF]
